# Women in Intensive Care study: a preliminary assessment of international data on female representation in the ICU physician workforce, leadership and academic positions

**DOI:** 10.1186/s13054-018-2139-1

**Published:** 2018-09-10

**Authors:** Bala Venkatesh, Sangeeta Mehta, Derek C. Angus, Simon Finfer, Flavia R. Machado, John Marshall, Imogen Mitchell, Sandra Peake, Janice L. Zimmerman

**Affiliations:** 1 Intensive Care, Wesley and Princess Alexandra Hospitals, Brisbane, QLD Australia; 2 College of Intensive Care Medicine, Prahran, VIC Australia; 3 The George Institute for Global Health, University of New South Wales, Sydney, NSW Australia; 4 Department of Medicine and Interdepartmental Division of Critical Care Medicine, Sinai Health System, University of Toronto, Toronto, ON Canada; 5 The Clinical Research, Investigation, and Systems Modeling of Acute illness [CRISMA] Center, Pittsburgh, PA USA; 6 Department of Critical Care Medicine, University of Pittsburgh School of Medicine, Pittsburgh, PA USA; 7 Royal North Shore Hospital, Sydney, NSW Australia; 8 Anesthesiology, Pain and Intensive Care Department, Federal University of São Paulo, Sao Paulo, SP Brazil; 9 Department of Surgery and Critical Care Medicine, St. Michael’s Hospital, Toronto, ON Canada; 10 Australian National University Medical School, Canberra, ACT Australia; 11 Department of Intensive Care Medicine, The Queen Elizabeth Hospital, University of Adelaide, Adelaide, SA Australia; 12 World Federation of Societies of Intensive and Critical Care Medicine, Houston, Texas USA; 13 Societies of Intensive and Critical Care Medicine, Houston Methodist Hospital, Houston, TX USA

**Keywords:** Critical care, Female, Gender, Intensive care, Representation, Women, Workforce

## Abstract

**Background:**

Despite increasing female enrolment into medical schools, persistent gender gaps exist in the physician workforce. There are limited published data on female representation in the critical care medicine workforce.

**Methods:**

To obtain a global perspective, societies (*n* = 84; 79,834 members (40,363 physicians, 39,471 non-physicians)) registered with the World Federation of Societies of Intensive and Critical Care Medicine were surveyed. Longitudinal data on female trainee and specialist positions between 2006-2017 were obtained from Australia and New Zealand. Data regarding leadership and academic faculty representation were also collected from national training bodies and other organisations of critical care medicine.

**Results:**

Of the 84 societies, 23 had a registered membership of greater than 500 members. Responses were received from 27 societies (*n* = 55,996), mainly high-income countries, covering 70.1% of the membership. Amongst the physician workforce, the gender distribution was available from six (22%) participating societies—mean proportion of females 37 ± 11% (range 26–50%). Longitudinal data from Australia and New Zealand between 2006 and 2017 demonstrate rising proportions of female trainees and specialists. Female trainee and specialist numbers increased from 26 to 37% and from 13 to 22% respectively. Globally, female representation in leadership positions was presidencies of critical care organisations (0–41%), representation on critical care medicine boards and councils (8–50%) and faculty representation at symposia (7–34%). Significant gaps in knowledge exist: data from low and middle-income countries, the age distribution and the time taken to enter and complete training.

**Conclusions:**

Despite limited information globally, available data suggest that females are under-represented in training programmes, specialist positions, academic faculty and leadership roles in intensive care. There are significant gaps in data on female participation in the critical care workforce. Further data from intensive care organisations worldwide are required to understand the demographics, challenges and barriers to their professional progress.

**Electronic supplementary material:**

The online version of this article (10.1186/s13054-018-2139-1) contains supplementary material, which is available to authorized users.

## Background

In the last 50 years, there has been a significant increase in the proportion of women training to be doctors such that the gender gap that had existed for centuries has largely disappeared. Indeed, in 1994, for the first time in the history of medical education in Canada, more women than men enrolled in first-year programmes at Canada’s 16 medical schools [1]. Similar trends have been reported in the USA, Australia and many European countries [2–4]. This trend is not limited to high-income countries, but is increasingly reflected across the globe [5, 6].

Despite increasing women graduates, persistent gender gaps exist in the physician workforce, and in particular in some specialties [7–10] and in positions of leadership in all specialties [11]. Female medical graduates report that they specifically do not choose certain specialties such as critical care medicine (CCM) as their first choice [12].

There are limited published data on the proportion of women specialists (attending) practising CCM. Workforce surveys from the USA, the UK and Australia report that the proportion of female specialist CCM physicians ranges from 14 to 26% [13–15]. A headcount of intensive care specialists in the UK in 2012 reported that 17% were women, although the figure rose to 26% when the data were examined in terms of the proportion of full-time equivalents [14]. Data from Australia suggest that although there has been a gradual increase in the female participation rate in CCM (about 17%), it still lags behind the proportion of female medical graduates and female medical specialists (27%) [15].

Whether these proportions of women in CCM are reflected globally is unknown. Data on numbers of women intensivists are limited by multiple factors. The bulk of the data originate from high-income countries [13–15]. Most of the published data relate to physicians who have completed training. Whilst there are systematic records of trainee demographics in certain regions of the world such as the UK, Australia and other high-income countries, there are insufficient data on the proportions of female CCM trainees in many regions. Besides, the point where the drop in the female representation occurs is unclear—at medical school or at base residencies. There is also a paucity of international data on the representation of women in academic and leadership positions in CCM.

Given that there are huge knowledge gaps around both the magnitude of the gender disparity issue and the potential sources or events that might be targeted to increase female representation, we sought to understand the availability of international data upon which we could build a framework for inquiry and intervention. Therefore, we undertook a study to gather information from across the globe on the representation of women in the CCM physician workforce.

The aims of this study included the following:

1. a survey of societies registered with the World Federation of Societies of Intensive and Critical Care Medicine (WFSICCM) [16] to determine the representation and demographic profile of the female physician workforce in intensive care;
2. data collection from national training bodies and other organisations of CCM pertaining to gender distribution in the following areas:
  intensive care trainees and specialists,
  leadership positions in the specialty since 2000 using presidency of international and national societies as an example,
  representation on the councils or boards of various CCM societies,
  faculty speaker representation at annual scientific meetings in the last 3 years using four leading symposia in different geographic regions as examples—annual scientific meetings of the Society of Critical Care Medicine (SCCM, USA), the European Society of Intensive Care Medicine (ESICM, Europe), the International Symposium of Intensive Care and Emergency Medicine (ISICEM, Belgium) and the College of Intensive Care Medicine (CICM, Australia and New Zealand); and
3. identifying gaps in data and knowledge.

## Methods

Data for this study were gathered from five sources:

- A survey of the member societies of the WFSICCM.
- Data from the College of Intensive Care Medicine (CICM) [14] of Australia and New Zealand (an established training body) to obtain longitudinal information on the composition of the trainee and specialist workforce, as not all international and national societies are training bodies.
- Data from published reports pertaining to gender representation in CCM.
- Data from personal correspondence with individual office bearers of international and national societies, including presidency of CCM societies since 2000 (see list under Acknowledgements) and national training and certifying bodies.
- Data on faculty representation at four leading international annual scientific meetings between 2015 and 2017.

### WFSICCM survey

The WFSICCM was established in 1977 and is a membership organisation comprised of national societies of intensive and critical care medicine. The WFSICCM now has a membership of 84 national professional societies that includes intensive and critical care practitioners (physician, nursing and allied health) throughout the world [16].

Over an 8-week period, between 1 July 2017 and 31 August 2017, all of the national societies (*n* = 84) registered with the WFSICCM were invited to participate in an online survey to examine proportional gender representation in CCM using the Survey Monkey tool. Invitations were sent to societies in the Americas (*n* = 21), Europe (*n* = 24), Asia (*n* = 28), Africa (*n* = 10) and Oceania (*n* = 1). To obtain maximal participation, all societies were notified of the survey in a cover letter from the President of the WFSICCM. Two reminders were sent to all societies during the 8-week survey period. The study was supported by the CICM and endorsed by the WFSICCM. The questionnaire is presented in Additional file 1.

### The CICM of Australia and New Zealand

The CICM is the sole body responsible for intensive care medicine specialist training and education in Australia and New Zealand [17]. The CICM has collected detailed demographic data by gender since the commencement of its training programme. We obtained data on the proportion of female trainee applicants, enrolments, the proportions of trainees undertaking part-time and deferred training and the number of Fellows graduating from the CICM from 2006. The use of data from the database was approved by the CICM.

The following definitions were used to report data from the CICM database: trainee, a doctor who is registered with the CICM training programme to become an intensive care specialist; Fellow, a doctor who has completed all of the requirements of the training programme with the CICM and is an intensive care specialist and a Fellow of the CICM; and specialist (attending), a fully trained CCM practitioner working as a consultant or as an attending in intensive care. Part-time training is defined as any trainee who applies to undertake a fraction of the commitment of a full-time trainee, including both in-hours and out-of-hours duties, as long as it is a minimum of 0.4 of a full-time equivalent. Deferred training is defined as any trainee who chooses to interrupt their training for parental, travel or alternative study purposes.

The proportions of female and male trainees undergoing part-time and deferred training were expressed as a percentage of the total number of trainees undertaking part-time or deferred training.

## Results

### WFSICCM survey

**Fig. 1:**
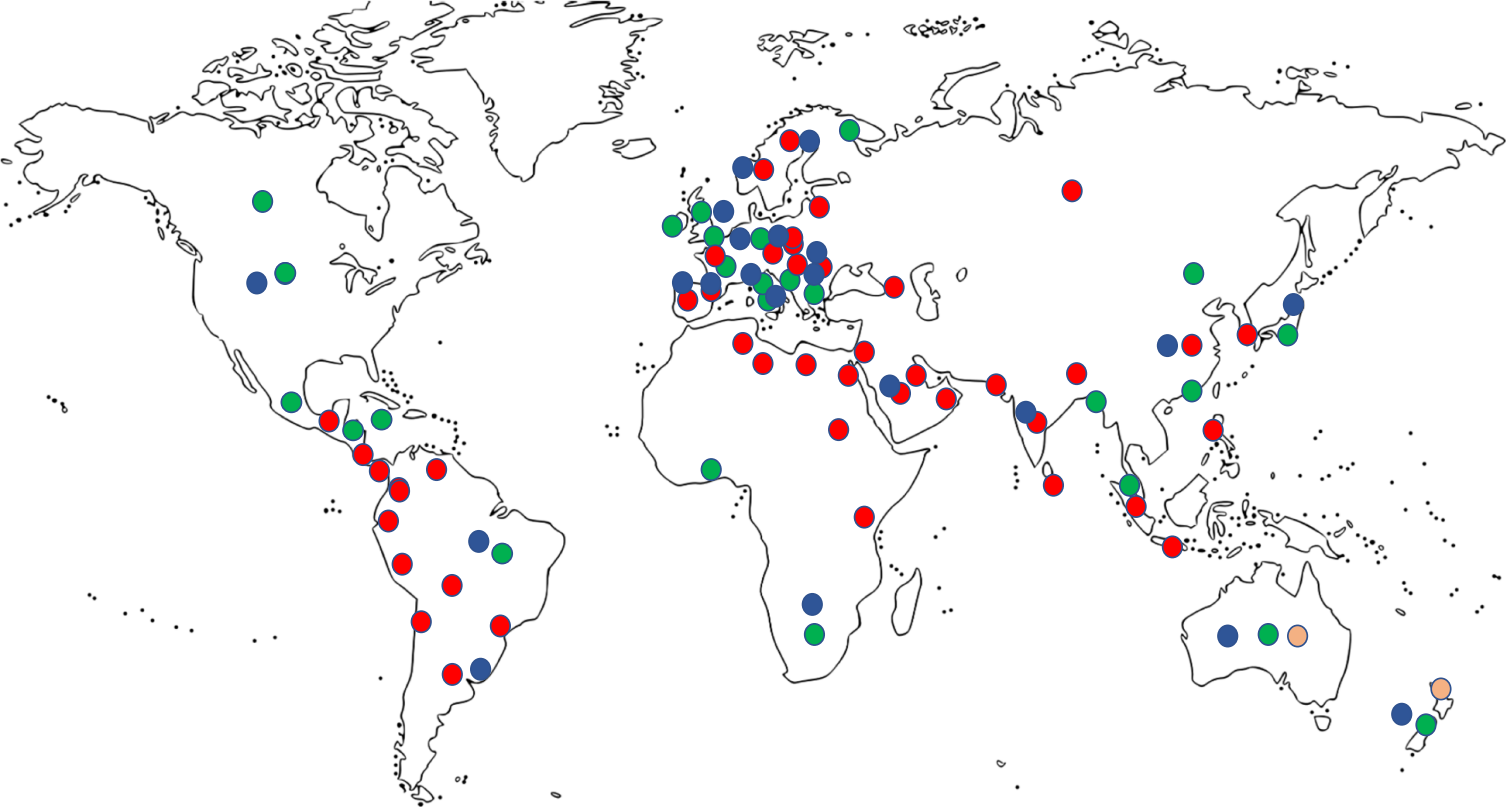
Map of various critical care societies registered with WFSICCM. Regions where societies responded to the WFSICCM survey in green. Survey data not available from regions in red. Areas in orange are those from which precise longitudinal data on gender representation were available. Areas in blue indicate regions with more than 500 registered members in the society

The 84 societies invited to participate in the survey have a total individual membership of 79,834. Responses were received from 27 societies (32.1%), covering a membership of 55,996 (70% of the total membership). The geographic distribution of the respondents was America 30%, Europe 37%, Asia 22%, Africa 7% and Oceania 4% (Fig. 1) and they were predominantly from high-income countries. Eighteen of the 27 responding societies (69%) were inter-professional (physician, nursing and allied health). The total number of physicians registered with the societies who responded was 40,259 (interprofessional 31,593, non-interprofessional 8666).

#### Gender distribution

Data on the gender distribution were available from 19 (14 interprofessional and five non-interprofessional; 51,248 members, 65%) of the 27 respondents. Amongst the physician workforce, data on gender distribution were available from six (22%) of the 27 participating societies (Australia and New Zealand, Guatemala, Italy, the Netherlands, Slovenia and the UK). The data from these six societies (9790 registered members) were pooled together to generate estimates of female ICM physician representation. The mean (SD) proportion of women across the six societies was 37 ± 11% (range 26–50%).

#### Age distribution and years since graduation

Only three societies collected data on age distribution and two societies on years since graduation. Consequently, data on the age distribution of females in the workforce and the time taken for women to achieve ICM specialist status since graduation was insufficient to report.

#### Proportion of female trainees

Only 30% of the survey respondents indicated that they were the national certifying bodies for intensive care training and assessment, and the distribution of data by gender was not available amongst those societies which reported the proportions of trainees.

The various society representatives highlighted areas of gender-related issues in intensive care requiring further study—gender disparities in pay, availability of maternity leave, the disproportionate number of night shifts given to women and minimal representation of women in leadership roles.

### Australia and New Zealand CCM trainee and specialist data: a longitudinal analysis

**Fig. 2:**
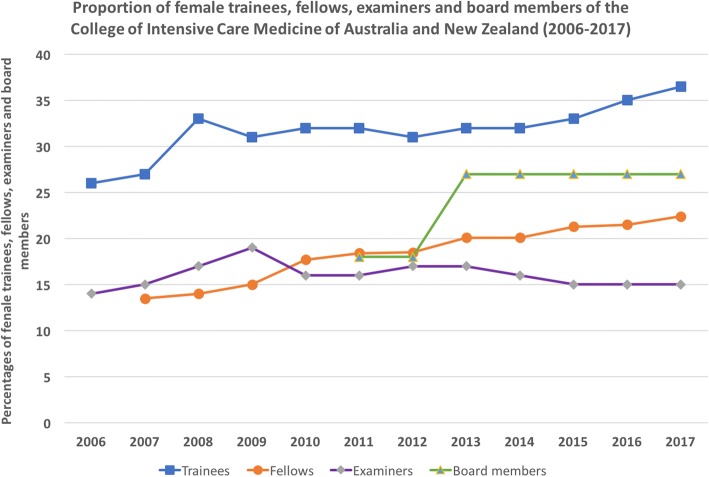
Illustration of proportions of female trainees, Fellows, examiners and board members of the College of Intensive Care Medicine of Australia and New Zealand between 2006 and 2017

**Fig. 3:**
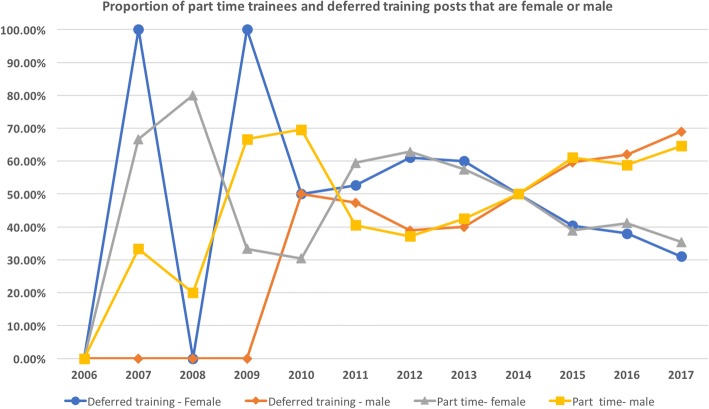
Proportion of part-time trainees and deferred training posts of the College of Intensive Care Medicine of Australia and New Zealand who are female or male between 2006 and 2017

Longitudinal data for trainees, Fellows, examiners and board representation by gender were available from the database of the Australian and New Zealand College of Intensive Care Medicine (CICM) between 2006 and 2017. During that period, there was a steady increase in the proportions of female trainees (from 26 to 37%) and Fellows (from 13 to 22%) (Fig. 2). The percentages of females and males undertaking part-time or deferred training expressed as a proportion of the total number of trainees undertaking part-time or deferred training fluctuated annually and appeared to be similar across both genders (Fig. 3).

Only Fellows of the CICM are eligible to be appointed as examiners or elected to the board. The proportion of female examiners has remained steady at around 15% over the 10-year period, whilst the proportion of female representation on the board of the college (formed in 2010) has ranged between 18 and 27% (Fig. 2).

### Summary of data from published reports and personal correspondence

A snapshot of female gender representation as trainees and specialists (consultants or attending) from different geographic regions of the world is presented in Table 1.

**Table 1:** Pooled data on female trainees and specialist representation, and membership of critical care societies, in intensive care medicine from different geographic regions

Region/country	Time period	Proportion of female trainees/specialists in the workforce
North America		
Canada (https://healthmanagement.org/c/icu/news/lives2017-is-there-a-critical-care-gender-gap) [32]	2015–2016	26.2% specialists 35% University of Toronto Paediatric and Adult CCM faculty
USA (https://healthmanagement.org/c/icu/news/lives2017-is-there-a-critical-care-gender-gap)	2011–2015	40% physicians who wrote American Board of Internal Medicine CCM certification examination
South America		
Argentina [33]	2010	43% physicians who completed CCM training
Brazil [34]	2012	30.2% of specialists in ICM were women
Europe		
France (https://healthmanagement.org/c/icu/news/lives2017-is-there-a-critical-care-gender-gap)	2012–2016	35% critical care trainees
Ireland [35] ^a^	2015	31.7% of specialists were women
Scandinavia (https://healthmanagement.org/c/icu/news/lives2017-is-there-a-critical-care-gender-gap)	2001–2017	42% enrolled in Scandinavian European Diploma in CCM programme
Spain [36]	2014	Femininity index^b^ in critical care medicine 0.08
UK (https://healthmanagement.org/c/icu/news/lives2017-is-there-a-critical-care-gender-gap)	2015	33% physicians practising anaesthetics/CCM; 41% in age < 40 years group
Asia		
China	2017	46% Chinese Society of CCM members
India^c^	2015–2017	32% trainees taking CCM—Part 2 examination 20% college of CCM members
Israel [37] ^a^	2011	22% of specialists were women
Africa		
Mozambique, Guinea and Cape Verde [38]^d^	2015	60% anaesthesiology and 29% internal medicine
Oceania		
New Zealand [39]	2012	30% of trainees and 18% of specialists were women.

In regions where clear numbers of trainees and specialists were not available, we have provided other relevant data relating to female participation in the ICM workforce. *CCM* critical care medicine

^a^In a number of countries, intensive or critical care medicine does not exist as an independent specialty. Patients are often managed by anaesthesiologists or internists and those figures have been reported when intensive or critical care medicine were not explicitly stated in the reports

^b^Ratio between the number of women and men in permanent medical positions in the specialties throughout the study period

^c^Personal correspondence with Dr Praveen Jain, Chairman, College of Critical Care Medicine, India

^d^Grouped data reported for the three countries

### Leadership positions within the specialty

Using presidency of major critical care organisations since the year 2000 as an example, we identified that female representation was very limited, ranging from 0 to 41% (Table 2). Female representation on various CCM boards and councils ranged from 8 to 50%, depending on the geographic region (Table 3). The society with the highest female representation (SCCM) on its board is interprofessional.

**Table 2:** Presidency of various international societies and bodies in intensive care

Society	Number (%) of female presidents 2000–2017
ESICM^a^	0/9 (0%)
SCCM (http://www.sccm.org/About-SCCM/Leadership/Past-Presidents)	7/17 (41%)
ANZICS (http://www.anzics.com.au/www.anzics.com.au/about-us.html)	1/9 (11%)
WFSICCM	1/5 (20%)
CICM of Australia and New Zealand^b^ (http://www.cicm.org.au/About/Honours-Awards#PastPresidentsandDeans)	0/5 (0%)

*ANZICS* Australia and New Zealand Intensive Care Society, *CICM* College of intensive Care Medicine, *ESICM* European Society of Intensive Care Medicine, *SCCM* Society of Critical Care Medicine, *WFSICCM* World Federation Society of Intensive and Critical Care Medicine

^a^Personal correspondence with Prof. Andrew Rhodes, past president of ESICM

^b^ Data for CICM only from 2010, the date of inception of the college

**Table 3:** Proportion of women on the council or board of national intensive care societies from different parts of the world

Society	Proportion of women in the council or board in 2017 (%)
North America	
SCCM (http://www.sccm.org/About-SCCM/Leadership/Council)	50
Canadian Critical Care Society (http://www.canadiancriticalcare.org/Governance)	36
South America	
Brazil (http://www.amib.org.br/diretoria/diretorias-anteriores/)	40
Europe	
ESICM [40]	8
Asia	
Chinese Society of Critical Care Medicine (Standing Committee of the 4th Committee of the Chinese Medical Association Critical Illness Branch) [41]	18
Sri Lankan Society of Critical Care and Emergency Medicine (http://www.ssccem.com/executive-committee)	21
Africa	
Critical Care Society of South Africa (http://www.criticalcare.org.za/About/Council)	29
Oceania	
ANZICS (http://www.anzics.com.au/www.anzics.com.au/about-us.html)	14
World Federation	
WFSICCM (http://www.world-critical-care.org/index.php?option=com_content&view=article&id=3&Itemid=3)	7

*ANZICS* Australia and New Zealand Intensive Care Society, *ESICM* European Society of Intensive Care Medicine, *SCCM* Society of Critical Care Medicine, *WFSICCM* World Federation Society of Intensive and Critical Care Medicine

### Proportion of female faculty at leading ICM symposia (2015–2017)

A review of the faculty representation over 3 years at the four leading ICM symposia from four different geographic regions revealed that women constituted only 7–34% of the total faculty (Table 4).

**Table 4:** Academic representation in major scientific meetings: proportion of female faculty

Meeting	2015 (%)	2016 (%)	2017 (%)
ESICM	15	15	16.9
SCCM^a^	29	30	27
ISICEM	7.5	11.4	7.8
CICM of Australia and New Zealand	7.7	17.2	34

*CICM* College of Intensive Care Medicine, *ESICM* European Society of Intensive Care Medicine, *ISICEM* International Society of Intensive Care and Emergency Medicine, *SCCM* Society of Critical Care Medicine

^a^SCCM includes a proportion of non-physician participants

The significant gaps in data and knowledge with regard to the female representation in the CCM physician workforce are summarised in Table 5.

**Table 5:** Gaps in knowledge and the potential usefulness of future research in this area

Gap in data	Significance
Precise estimates of female representation in CCM trainee and specialist workforce worldwide Age distribution of the male and female workforce Time taken to complete CCM training Proportion of women having to undertake part-time or deferred training Drop-out rates from CCM training Proportion of women undertaking fractional appointments in intensive care as a specialist	Facilitate planning for workforce and gender diversity Major differences in age distribution, may point to factors such as family/home commitments which are predominantly borne by women Prolonged training times for women may be an indicator of women having to undertake part-time training owing to other commitments Will enable training bodies to introduce flexible training options May be an indicator of competing family and domestic responsibilities and lack of roster-friendliness, provide alternative rostering options

## Discussion

The principal finding of this study is that there is limited information on female representation in the intensive care physician workforce in the various national intensive care society registries. The precise estimate of the female physician workforce is further limited by the interprofessional nature of many societies. Longitudinal data from a regional training body suggest a rising trend in the proportion of women in the workforce, and the proportions of trainees undertaking part-time training or having to defer their training appeared to be similar across both genders. There was a significant under-representation of women as faculty in major scientific meetings and in leadership roles of major ICM organisations. These findings are in accord with published data from other specialties of medicine [7–10, 15].

### Potential reasons for under-representation of women in the ICU workforce

Although our study did not investigate the reasons for the reduced representation of women in the CCM workforce, possible explanations include the following. Until recently, training in intensive care medicine in many countries was undertaken after completion of a primary specialty pathway such as internal medicine, anaesthesia or emergency medicine, which had implications for duration of training and consequently on lifestyle and career choices, confirmed in a recent study of internal medicine residents [18] and medical students [19] and a survey of female intensive care specialists in Australia [20]. Other findings reported in the Australian survey were the prevalence of unprofessional behaviours such as sexism and discrimination in the workplace and difficulties with academic advancement [20]. These reports are in accord with data from a recent publication by Mehta et al. [21], and a recent survey of intensive care trainees and Fellows from Australia and New Zealand also reported that females were most likely to experience sexual harassment and discrimination [22]. In addition, the reasons behind career choices are often complex and the choice of a specialty is often influenced by the degree of exposure to the specialty at various stages of training (medical student or as a junior trainee or resident) and the motivational effect of role models and mentors. Another potential factor is the stressful nature of the ICU work environment reported in a recent survey of intensive care physicians in India, where women reported higher levels of stress [23].

Our study has also identified that despite women representing anywhere between 20 and 40% of the CCM specialist workforce depending on the geographic region, their proportional representation in leadership and academic positions is lower. Whether this is a chance observation or a real finding cannot be deduced from our survey. Studies from other areas of medicine have reported gender-based differences in career advancement [24], leadership positions [11], byline positions in the case of dual first authorship [25] and salary [26]. Similar concerns were highlighted by our survey respondents as areas for future research. These findings together with other published data raise the possibility of conscious and unconscious bias against female physicians in general and should be the subject of future investigations.

There has been increasing awareness of gender disparity in intensive care medicine and training bodies, and organisations have responded to the call for action with the introduction of flexible training policies (including part-time and interrupted training, and parental leave options), guidelines for professional standards of behaviour and for appropriate gender representation at various events, and promotion of diversity and equality (https://www.cicm.org.au/Trainees/Training-FAQ, http://cicm.org.au/CICM_Media/CICMSite/CICM-Website/Resources/Professional%20Documents/IC-20-Prevention-of-Bullying-Discrimination-and-Harassment-in-the-Workplace_1.pdf, https://www.cicm.org.au/CICM_Media/CICMSite/CICM-Website/Resources/Professional%20Documents/IC-3-Minimum-Standards-for-Intensive-Care-Units-Seeking-Accreditation_3.pdf, https://www.esicm.org/announcement-esicm-launches-diversity-task-force-march-2018/) [27]. Research granting bodies worldwide have developed gender equity policies [28, 29]. Development of policies to increase female representation may also potentially address the shortage of health professionals in CCM in both high and low-income countries [30, 31].

The strengths of our study include provision of a snapshot of the female physician workforce from our survey of both high and low-income countries. In addition, we were able to report data for a longitudinal study from a well-established training body, which is the sole provider of training in intensive care medicine in that region, thus minimising confounders from parallel training pathways seen in other parts of the world. We have also identified key gaps in knowledge and areas for targeted research in the future (Table 5).

Our survey had limitations. We surveyed national societies registered with the WFSICCM. A substantial proportion of these societies were not training bodies and it is possible that data about female intensive care trainees and specialists registered only with the training bodies but not with the national society may not have been captured by this survey. Although there was a limited response rate from the various societies, the target population of the respondents covered nearly 70% of the total individual membership. The responses were also largely from high-income countries, and specifically data on the female physician workforce was only available from six countries; consequently, the demographic profile and sex distribution may not be representative of the global CCM community. We attempted to collect data on the times taken for females to complete training in the specialty and the average age of appointment to a consultant/specialist position, but were limited by the availability of data from the various societies. As this was a preliminary survey, we did not capture data on the job stresses faced by female CCM physicians such as the balance between work and family demands, and sexism in the workplace.

In conclusion, there is limited information on female participation in the intensive care workforce. Available data suggest that females are under-represented in training programmes, specialist positions, academic faculty and leadership roles in intensive care. We recommend that national societies and training and certifying bodies collect more detailed demographic data of their membership, engage with training bodies to promote female enrolment into training programmes, facilitate part-time training and work with employers to develop policies to minimise bullying, discrimination and sexual harassment behaviours in the workplace. Further studies are required to capture data from all of the international training bodies to obtain precise numbers of women trainees and specialists in intensive care worldwide and to understand the challenges and the barriers to their professional progress.

## Conclusion

Despite limited information globally, available data suggest that females are under-represented in training programmes, specialist positions, academic faculty and leadership roles in intensive care. There are significant gaps in data on female participation in the critical care workforce. Further data from intensive care organisations worldwide are required to understand the demographics, challenges and barriers to their professional progress.

## Additional file

Additional file 1: WFSICCM survey template. (DOCX 16 kb)

## Abbreviations

CCM: Critical care medicine
CICM: College of intensive Care Medicine
ESICM: European Society of Intensive Care Medicine
ISICEM: International Society of Intensive Care and Emergency Medicine
SCCM: Society of Critical Care Medicine
WFSICCM: World Federation Society of Intensive and Critical Care Medicine
