# Supplementary material for: Women in Intensive Care study: a preliminary assessment of international data on female representation in the ICU physician workforce, leadership and academic positions
Source: Crit Care. 2018 Sep 10;22:211. doi: 10.1186/s13054-018-2139-1 (PMC6130077; doi:10.1186/s13054-018-2139-1)
Supplement: Supplementary file 1 — WFSICCM survey template. (DOCX 16 kb) [file 13054_2018_2139_MOESM1_ESM.docx]

**WFSICCM survey template**

1. Name of the Society.
2. Are you an Interprofessional Society? Yes No

⃝ Physicians ⃝ Nurses ⃝ Allied Health (pharmacist, physiotherapist)

1. Country:
2. Name and position of the person completing this form.
3. Number of registered members in the Society: (complete if applicable

Total number:

Doctors:

Nurses:

Allied Health:

1. Gender distribution of members:

⃝ We do not have this information

Total:

Doctors:

Nurses:

Allied Health:

1. Age distribution of members if available:

⃝ We do not have this information

Doctors Nurses Allied health

M/F M/F M/F

25-29

30-39

40-49

50-59

>60

1. Years since graduation

⃝ We do not have this information

Doctors Nurses Allied health

M/F M/F M/F

< 5

6-10

11-15

16-20

21-30

31-40

>40

1. What proportion of the doctors are
2. Trainees Males/Females
3. Specialists/Attending Males/Females
4. Is your organization also the National Certifying Body for Critical Care? Yes/No
5. If you responded ‘No’ to Q10, do you have a National Certifying Board / College/Assessment Examination for Critical Care? Yes No. If yes, please state the

Name of the body:

Name and contact details of the Administrative officer:

1. We are proposing to do a future study / studies exploring demographics and gender issues in intensive care. This may take the form of a survey(s) of individual members of your society. Are you or any of your colleagues willing to be the regional coordinator for such a survey? Yes No.
2. If Yes to 12, can you provide an email address? (Free text)
3. If your organization is willing to take part in future surveys, would you be willing to share any other anonymized spreadsheets or databases, which may provide further insight into demographic and gender distribution in intensive care medicine? Yes No
4. If there is a follow on survey, we would like your feedback on other gender related issues you think are important and worth exploring? (Free text)
5. What in your opinion would be the best method of conducting the survey?

- ⃝ paper survey
- ⃝ Using internet tools such as a survey monkey
